# Self-reported symptoms as predictors of SARS-CoV-2 infection in the general population living in the Amsterdam region, the Netherlands

**DOI:** 10.1371/journal.pone.0262287

**Published:** 2022-01-28

**Authors:** Jizzo R. Bosdriesz, Feiko Ritsema, Tjalling Leenstra, Mariska W. F. Petrignani, Sylvia M. Bruisten, Liza Coyer, Anja J. M. Schreijer, Yvonne T. H. P. van Duijnhoven, Maarten F. Schim van der Loeff, Amy A. Matser

**Affiliations:** 1 Department of Infectious Diseases, Public Health Service of Amsterdam, Amsterdam, The Netherlands; 2 Department of Internal Medicine, Division of Infectious Diseases, Amsterdam UMC, University of Amsterdam, Amsterdam, The Netherlands; 3 Department of Medical Microbiology, Amsterdam Infection & Immunity Institute (AII), Amsterdam University Medical Center (UMC), Amsterdam, The Netherlands; Seoul National University College of Medicine, REPUBLIC OF KOREA

## Abstract

**Introduction:**

Most COVID-19 symptoms are non-specific and also common in other respiratory infections. We aimed to assess which symptoms are most predictive of a positive test for SARS-CoV-2 in symptomatic people of the general population who were tested.

**Methods:**

We used anonymised data of all SARS-CoV-2 test results from the Public Health Service of Amsterdam from June 1,2020 through August 31, 2021. Symptoms were self-reported at time of requesting a test. Multivariable logistic regression models with generalized estimating equations were used to identify predictors of a positive test. Included symptoms were: cough, fever, loss of smell or taste, muscle ache, runny nose, shortness of breath, and throat ache; adjustments were made for age and gender, and stratification by month.

**Results:**

Overall, 12.0% of 773,680 tests in 432,213 unique individuals were positive. All symptoms were significantly associated with a positive test result, the strongest positive associations were: cough (aOR = 1.78, 95%CI = 1.75–1.80), fever (aOR = 2.11, 95%CI = 2.07–2.14), loss of smell or taste (aOR = 2.55, 95%CI = 2.50–2.61), and muscle ache (aOR = 2.38, 95%CI = 2.34–2.43). The adjusted odds ratios for loss of smell or taste slightly declined over time, while that for cough increased.

**Conclusion:**

Cough, fever, loss of smell or taste, and muscle ache appear to be most strongly associated with a positive SARS-CoV-2 test in symptomatic people of the general population who were tested.

## Introduction

Starting in December 2019, the world has experienced the emergence of the novel severe acute respiratory syndrome coronavirus 2 (SARS-CoV-2), quickly reaching nearly every country on the globe. SARS-CoV-2 causes coronavirus disease 2019 (COVID-19), which in March 2020 the WHO declared a pandemic [1]. As of November 2021, there have been over 240 million confirmed cases reported worldwide, resulting in over 5 million confirmed deaths [1].

Symptomatology of SARS-CoV-2 infection may vary significantly in nature and severity, from no symptoms at all, mild symptoms, to life-threatening symptoms requiring intensive care [2]. Pooled estimates from meta-analyses indicate that around 25% of people who test positive remain asymptomatic [3–5]. There is mixed evidence on whether the percentage of asymptomatic infections for the emerging variants of concern is different from the wildtype [6]. For patients who exhibited symptoms, the most commonly reported symptoms of infection with the wild type SARS-CoV-2 were: fever, cough, fatigue, muscle ache (myalgia), shortness of breath (dyspnoea) or chest pain, loss of smell or taste (anosmia/ageusia), and other upper respiratory tract complaints [7, 8]. There is currently no consensus on whether the symptoms associated with infection with variants of concern such as the Alpha or Delta variants are different from those reported for the wild type [9, 10].

Many of these reported symptoms are also commonly found in other diseases. For example fever, cough, sore throat, and muscle ache are among the most often reported symptoms of influenza [11], whereas sore throat, runny nose (rhinorrhoea), sneezing, nasal congestion, and cough might also be indicative of a common cold or allergic rhinitis (hay fever) [12, 13]. One thing these highly prevalent respiratory conditions have in common, is that their incidence typically shows a seasonal pattern, where allergic rhinitis (caused by grass and tree pollen) is most prevalent during spring and summer, and the common cold and influenza are more typically found in winter [14–16]. This overlap between symptoms of COVID-19 and other common respiratory conditions makes it hard to predict a diagnosis of COVID-19 based on the presence or absence of certain symptoms. Moreover, allergic disorders of the upper respiratory tract might also be a risk factor for COVID-19 [17, 18].

Loss of smell and taste appear to be symptoms specific to COVID-19, rarely occurring in other respiratory infections. Nevertheless these may only occur in 4% to 25% of COVID-19 cases [7], and therefore their absence does not rule out COVID-19. Using a combination of symptoms might be more predictive, but so far evidence-based models using symptoms to predict a diagnosis of COVID-19 are lacking. A systematic review of COVID-19 prediction models identified four models that predict the risk in the general population (most other models used data of hospitalized patients), but they used demographics and medical history as predictors [19]. Some studies not included in the review also reported on prediction models. These were two studies among health care workers [20, 21], and two general population studies, one using a short online survey [22], and one using an app with real-time symptom tracking [8]. Loss of smell or taste was the only symptom to be consistently included in all of these models; some models also included: cough, shortness of breath, fever, fatigue, headache, and sore throat.

A better understanding of which symptoms or combinations of symptoms are most predictive of COVID-19 could inform SARS-CoV-2 testing policies. As a result of the rapid rise of cases during the first months of the pandemic, many countries including the Netherlands, put in place policies that were not evidence-based, since there was a lack of evidence and testing capacity was low. As the pandemic has progressed and the global knowledge of the virus and the disease expanded, it is prudent to refine those policies based on the emerging evidence. For instance, in situations where the capacity for laboratory testing for SARS-CoV-2 is limited or insufficient, a triaging system based on symptoms that are proven to be associated with diagnosis could be helpful to give priority to screening those who are most likely to be infected, improve the accuracy of testing, and prevent unnecessary spending [23]. In addition, many public spaces such as health care facilities, public transport, retail, dining and cultural venues have employed some form of brief symptom-based screening to prevent infectious people from entering and potentially spreading the virus to others [24]. For such an approach to be effective, it would need to be evidence-based, and screen for the most predictive symptoms. Moreover, in a situation of a low prevalence with occasional small and localized outbreaks, such a screening tool could be used to guide diagnostics and determine whether further measures are needed to contain the outbreak. In this way it could be integrated in existing sentinel surveillance networks of general practice data [25].

Therefore, the aim of this paper is to investigate which symptoms or combinations of symptoms are most predictive of a positive SARS-CoV-2 nucleic acid amplification technique (NAAT) among people from the general population who present themselves for testing. Due to the seasonal nature of the occurrence of respiratory conditions with overlapping symptoms (allergic rhinitis, common cold, influenza), in addition to the potentially changing profile of associated symptoms since the emergence of the Alpha and Delta variants, we additionally studied whether the associations between symptoms and SARS-CoV-2 test result changed over time.

## Materials and methods

### Population

The study population consisted of individuals who were tested in one of the publicly funded SARS-CoV-2 testing facilities affiliated with the Public Health Service (PHS) of Amsterdam, the Netherlands, from June 1^st^ 2020 through August 31^st^ 2021. From June 1^st^ 2020, every person who had symptoms suggestive of a SARS-CoV-2 infection could request a test by phone or online via the test request application. Since December 1^st^ 2020, close contacts of SARS-CoV-2 infected cases who have not developed symptoms within five days after their last exposure could request a test as well. Close contacts are people identified by PHS contact tracing or the Dutch Government Corona tracker app who have been within 1.5 meters of a confirmed case for more than cumulative 15 minutes. Since it is possible for people to be tested multiple times, there are potentially multiple records per person.

Duplicate records (same person, same date) were removed, as well as records with indeterminate or inconclusive test results and records with missing data on date of birth or gender. In addition, we removed all records with a testing date less than 31 days after a previous test. This is based on the National Institute for Public Health and the Environment’s guideline on risk for re-infection and re-testing [26]. Lastly, records in which no symptoms were reported (either for asymptomatic cases or missing data) were also excluded from the analyses, because the focus of this paper is to study the predictive value of symptoms.

### Data collection

For both test request routes, people requesting a test were asked to provide information on which symptoms they were currently experiencing, and the date of onset of symptoms. Date of birth and gender were obtained from the Dutch population registry. With these demographic and symptom-related data, a record was generated in CoronIT, the database system used by the Dutch PHS to collect and store data of individuals attending SARS-CoV-2 testing facilities. Nose and throat swabs were subsequently collected at one of the SARS-CoV-2 testing facilities, preferably within 24 hours of making the appointment. These swabs were tested at the laboratory of the PHS of Amsterdam or other contracted laboratories, using one of several NAAT methods, depending on the lab and period: transcription-mediated amplification (TMA), polymerase chain reaction (PCR), or loop-mediated isothermal amplification (LAMP). All test were added to the existing records in the CoronIT database. For this study, routine data were extracted retrospectively from CoronIT and anonymised.

As study data were collected retrospectively from the source database and the study population was very large, it was not possible to obtain informed consent. The study complies with the Dutch Law on the Medical Treatment Agreement (WGBO art. 7:458) and the European General Data Protection Regulation (art. 9.2.j & art. 89). The medical ethics committee of the Amsterdam University Medical Centers deemed the study outside the scope of the ‘Medical Research Involving Human Subjects Act’ (W20_432#20.479).

### Variables

The outcome variable is the SARS-CoV-2 test result, which is either positive, indicating the presence of infection, or negative, indicating no infection. Indeterminate test results were excluded from our dataset.

The symptoms evaluated as potential predictors were: cough, fever, loss of smell or taste, muscle ache, runny nose, shortness of breath, or throat ache; all symptom data were self-reported by the clients. Loss of smell and loss of taste were assessed separately but combined into one variable indicating the presence of either symptom. Additional variables include age in 10 year groups, and gender (male/female). The variable month was included to study changes over time, ranging from June 2020 through August 2021.

### Analyses

We listed the number and percentage of positive and negative test results by age, gender, calendar week, and presence of symptoms. Furthermore, we calculated the distribution (median and interquartile range [IQR]) of the sample in terms of age, and number of symptoms reported. For each symptom, we also calculated the negative predictive value (NPV), i.e. the probability that someone without the symptom will test negative; and the positive predictive value (PPV) i.e. the probability that someone with the symptom will test positive.

To analyse which symptoms were associated with test result, we used logistic regression models in which all symptoms were included, and adjusted for age and gender. Generalized estimating equations (GEE) were used to account for repeated testing. Additionally, we built separate models for each calendar month (without GEE) to assess whether the influence of symptoms on the test outcome changed over time. To test whether these changes were statistically significant, we built multivariable models with interaction terms for each symptom in separate models.

To assess if certain symptoms tend to occur in conjunction, we performed a principal components analysis (PCA), using varimax rotation with Kaiser normalization [27].

All descriptive analyses and regression analyses were performed in IBM SPSS Statistics Version 26.0.0.1.

## Results

In the study period, 1,260,614 tests were performed in the region of Amsterdam and analysed by the PHS. 134,961 (10.71%) records with missing, inconclusive or indeterminate test results were removed, as well as 2,669 (0.21%) records with missing age or gender, 37,384 (2.97%) duplicate records or records with a test date less than 31 days after the previous test date, and 311,920 (24.74%) records without symptoms (see S1 Fig). After these exclusions, the study sample consisted of 773,680 tests, in 432,213 unique individuals. There were more women in the sample than men (55.3% vs. 44.7%). The median age at time of testing was 33 years (IQR = 25–46), and the median number of reported symptoms was 2 (IQR = 1–3).

Overall, 92,486 (12.0%) SARS-CoV-2 test results were positive. The percentage of positive test results increased from 1.5% in June 2020 to 18.5% in October, decreasing again to 7.6% in March 2021, and increasing again to 20.5% in August 2021. Men had a slightly higher percentage of positive tests than women (12.8% vs. 11.3%), and the percentage of positive test results also varied somewhat by age, with the lowest rates seen in the 0–14 and 35–44 age groups, and the highest rates in the 15–24 age group. In addition, there was an upward trend in the percentage of positive tests by number of reported symptoms, from 9.0% for 1 symptom up to 21.0% for ≥4 symptoms. More detailed descriptive information on the study sample is shown in Table 1 and S1 Table.

**Table 1:** The number and percentage of positive SARS-CoV-2 test results by number of symptoms, month, gender, and age group, from June 2020 through August 2021, the Amsterdam region, the Netherlands.

	Total (N)	Negative (N)	Negative (%)	Positive (N)	Positive (%)
TOTAL	773,680	681,194	88.0%	92,486	12.0%
**Gender**					
Female	427,975	379,608	88.7%	48,367	11.3%
Male	345,705	301,586	87.2%	44,119	12.8%
**Age group (years)**					
0–14	61,861	57,342	92.7%	4,519	7.3%
15–24	130,542	109,148	83.6%	21,394	16.4%
25–34	227,031	201,711	88.8%	25,320	11.2%
35–44	148,096	133,798	90.3%	14,298	9.7%
45–54	91,972	79,730	86.7%	12,242	13.3%
55–64	66,327	57,235	86.3%	9,092	13.7%
65–74	35,062	31,103	88.7%	3,959	11.3%
75+	12,789	11,127	87.0%	1,662	13.0%
**Number of symptoms**					
1	258,137	234,799	91.0%	23,338	9.0%
2	265,238	236,612	89.2%	28,626	10.8%
3	159,559	138,104	86.6%	21,455	13.4%
≥4	90,746	71,679	79.0%	19,067	21.0%
**Month**					
June 2020	16,087	15,847	98.5%	240	1.5%
July 2020	25,350	24,734	97.6%	616	2.4%
August 2020	53,316	50,657	95.0%	2,659	5.0%
September 2020	65,041	57,624	88.6%	7,417	11.4%
October 2020	84,570	68,947	81.5%	15,623	18.5%
November 2020	60,152	51,949	86.4%	8,203	13.6%
December 2020	88,278	78,523	88.9%	9,755	11.1%
January 2021	48,395	43,092	89.0%	5,303	11.0%
February 2021	39,245	35,707	91.0%	3,538	9.0%
March 2021	82,732	76,407	92.4%	6,325	7.6%
April 2021	66,825	59,754	89.4%	7,071	10.6%
May 2021	43,068	38,092	88.4%	4,976	11.6%
June 2021	24,264	22,995	94.8%	1,269	5.2%
July 2021	53,273	38,518	72.3%	14,755	27.7%
August 2021	23,084	18,348	79.5%	4,736	20.5%

Of the seven studied symptoms, runny nose, throat ache, and cough were the most prevalent in the total sample (63.7%, 54.4%, and 44.2% respectively), as shown in Table 2. Loss of smell or taste was the least prevalent symptom at 8.6%, but this was around three times more prevalent in those who tested positive compared to those who tested negative. The negative predictive value (i.e. the probability that someone without the symptom will test negative) was quite high for all symptoms, ranging from 85.1% for runny nose to 90.6% for cough. The positive predictive value (i.e. the probability that someone with the symptom will test positive) was much lower and varied between symptoms, ranging from 10.0% for throat ache to 26.6% for loss of smell or taste.

**Table 2:** The prevalence of each symptom by SARS-CoV-2 test result and overall; and the negative and positive predictive value, from June 2020 through August 2021, the Amsterdam region, the Netherlands.

	Total		Negative		Positive		NPV (%)	PPV (%)
Prevalence	N	%	N	%	N	%		
Cough	342,155	44.2	290,400	42.6	51,755	56.0	90.6	15.1
Fever	144,941	18.7	113,525	16.7	31,416	34.0	90.3	21.7
Loss of smell or taste	66,691	8.6	48,958	7.2	17,733	19.2	89.4	26.6
Muscle ache	97,888	12.7	73,077	10.7	24,811	26.8	90.0	25.3
Runny nose	492,484	63.7	441,814	64.9	50,670	54.8	85.1	10.3
Shortness of breath	107,908	13.9	93,500	13.7	14,408	15.6	88.3	13.4
Throat ache	420,993	54.4	378,861	55.6	42,132	45.6	85.7	10.0

* NPV = negative predictive value (the probability that someone without the symptom will test negative). ** PPV = positive predictive value (the chance that someone with the symptom will test positive).

Table 3 shows the main results of the logistic regression models for the overall study sample. All predictors were significantly associated with a positive SARS-CoV-2 test, but the strongest positive associations were found for cough with an adjusted odds ratio (aOR) of 1.78, with a 95% confidence interval (CI) from 1.75 to 1.80, fever (aOR = 2.11, 95%CI = 2.07–2.14), loss of smell or taste (aOR = 2.55, 95%CI = 2.50–2.61), and muscle ache (aOR = 2.38, 95%CI = 2.34–2.43). Runny nose (aOR = 0.70, 95%CI = 0.69–0.71), shortness of breath (aOR = 0.74, 95%CI = 0.72–0.75), and throat ache (aOR = 0.60, 95%CI = 0.59–0.61) were associated with a negative test result. The area under the receiver operating characteristic (ROC) curve (AUC) was 0.72, indicating a relatively poor fit.

**Table 3:** Effect size and importance per symptom in the multivariable models for a positive SARS-CoV-2 test outcome, from June 2020 through August 2021, the Amsterdam region, the Netherlands.

	Model 1		
	aOR	95% CI	
Cough	**1.78**	1.75	1.80
Fever	**2.11**	2.07	2.14
Loss of smell or taste	**2.55**	2.50	2.61
Muscle ache	**2.38**	2.34	2.43
Runny nose	**0.70**	0.69	0.71
Shortness of breath	**0.74**	0.72	0.75
Throat ache	**0.60**	0.59	0.61

Model 1: Multivariable logistic regression model using generalized estimating equations including all symptoms, adjusted for age and sex. OR = odds ratio, 95% CI = 95% confidence interval. The AUC (Area under the receiver operating characteristic (ROC) curve) of Model 1 is 0.72.

**Fig 1:**
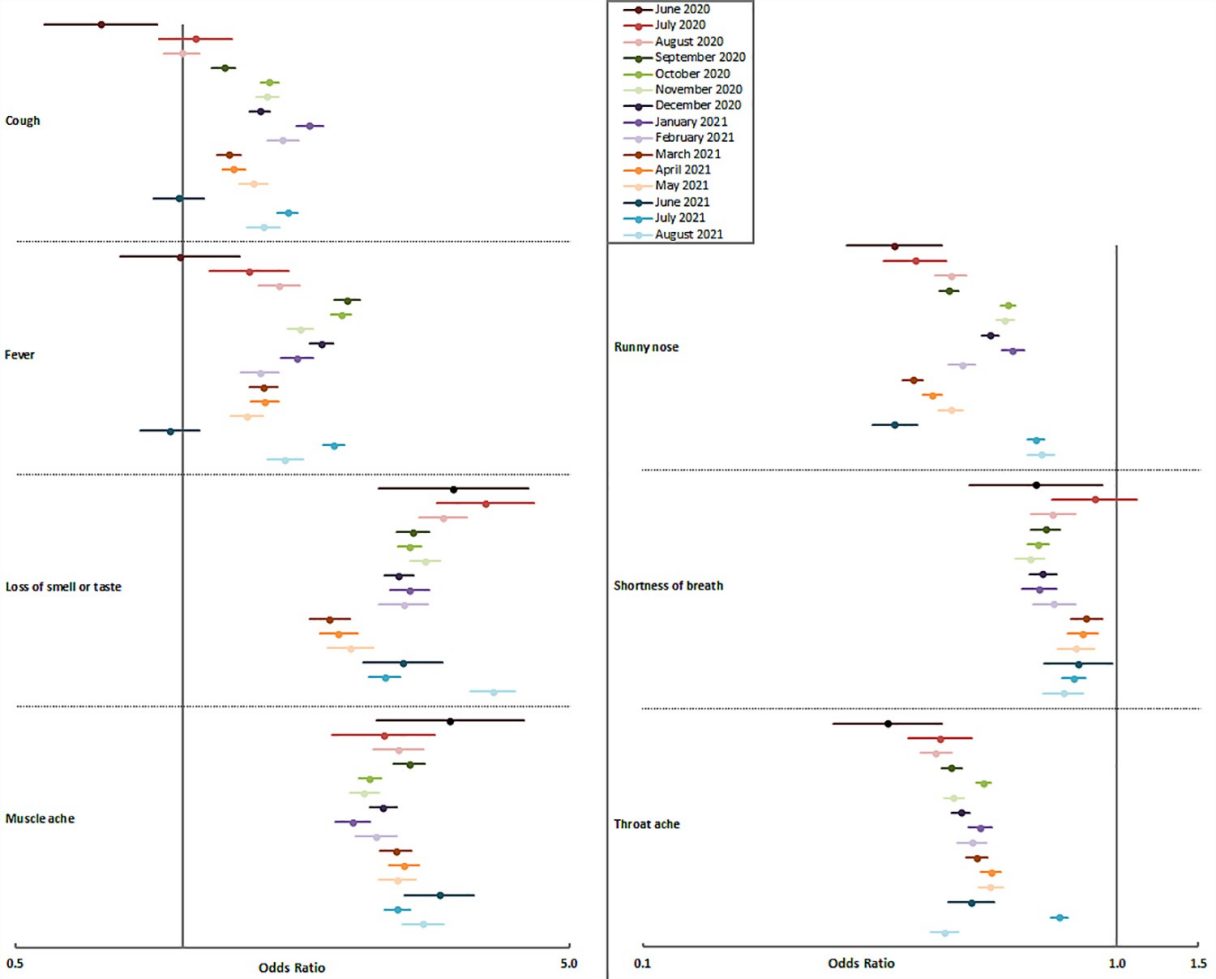
Odds ratios and 95% confidence intervals per variable in the full multivariable logistic regression model per month, from June 2020 through August 2021, the Amsterdam region, the Netherlands.

The results of the regression models per month are shown in Fig 1. Here, it can be seen that the aOR for loss of smell or taste seemed to decline slightly over time, whereas that for cough seemed to increase slightly. The aORs for the other symptoms remained mostly stable over time. The interaction models showed that the interaction between time and symptom were significant for each symptom except runny nose. The absence of pronounced differences in the models over time might be partly explained by the relatively stable prevalence of the individual symptoms over time (S2 Fig).

Table 4 shows the results of the logistic regression models per age group and by gender. The aOR for cough and fever increased with increasing age and then plateaued. The OR for loss of smell or taste, and muscle ache increased from the youngest age groups onward, peaked at 35–44 and 45–54 respectively, and then decreased again with increasing age. For these four symptoms, all ORs (except cough for 0–14 old) were above 1, and in most cases above 1.5. Runny nose, shortness of breath, and throat ache were stable across age groups, and below 1 in all cases. The stratified models by gender showed similar general patterns for men and women. The individual ORs for cough, loss of smell or taste, runny nose, and shortness of breath were slightly higher for women, whereas the ORs for fever, muscle ache, and throat ache were slightly higher for men.

**Table 4:** Odds ratios and 95% confidence intervals per symptom in full multivariable logistic regression models per age group and gender, from June 2020 through August 2021, Amsterdam region, the Netherlands.

	0–14 years			15–24 years			25–34 years			35–44 years			45–54 years		
	aOR	95% CI		aOR	95% CI		aOR	95% CI		aOR	95% CI		aOR	95% CI	
Cough	0.69	0.65	0.73	1.56	1.51	1.61	1.93	1.88	1.99	1.79	1.72	1.86	2.03	1.95	2.11
Fever	1.19	1.11	1.27	1.83	1.76	1.90	2.14	2.07	2.21	2.32	2.22	2.42	2.38	2.28	2.49
Loss of smell or taste	2.44	2.17	2.74	2.48	2.38	2.59	2.63	2.53	2.73	3.35	3.16	3.56	2.35	2.21	2.50
Muscle ache	2.14	1.90	2.41	1.88	1.80	1.97	2.32	2.24	2.40	2.74	2.57	2.92	2.77	2.65	2.91
Runny nose	0.55	0.52	0.59	0.78	0.75	0.80	0.77	0.74	0.79	0.63	0.61	0.66	0.63	0.60	0.65
Shortness of breath	0.83	0.73	0.94	0.80	0.76	0.83	0.79	0.76	0.83	0.75	0.70	0.79	0.66	0.62	0.70
Throat ache	0.69	0.64	0.73	0.67	0.65	0.69	0.66	0.65	0.68	0.51	0.49	0.54	0.57	0.55	0.59
	55–64 years			65–74 years			75 years and above			Females			Males		
	aOR	95% CI		aOR	95% CI		aOR	95% CI		aOR	95% CI		aOR	95% CI	
Cough	2.14	2.04	2.25	2.04	1.90	2.20	2.03	1.82	2.27	1.85	1.81	1.88	1.70	1.66	1.74
Fever	2.25	2.14	2.37	2.28	2.12	2.46	2.26	2.01	2.54	1.96	1.91	2.01	2.26	2.21	2.31
Loss of smell or taste	2.13	1.99	2.29	2.26	2.02	2.53	1.67	1.38	2.02	2.79	2.71	2.87	2.31	2.24	2.38
Muscle ache	2.47	2.34	2.61	2.43	2.24	2.65	1.60	1.39	1.84	2.32	2.27	2.38	2.46	2.40	2.53
Runny nose	0.65	0.62	0.68	0.67	0.62	0.71	0.63	0.56	0.70	0.72	0.71	0.74	0.66	0.65	0.68
Shortness of breath	0.62	0.58	0.67	0.65	0.59	0.71	0.71	0.62	0.81	0.77	0.75	0.79	0.70	0.68	0.72
Throat ache	0.58	0.55	0.60	0.57	0.53	0.61	0.65	0.58	0.73	0.59	0.58	0.60	0.62	0.60	0.63

All models by age group: Multivariable logistic regression model using generalized estimating equations that included all symptoms, adjusted for sex, modelled separately by age group. All models by sex: Multivariable logistic regression model using generalized estimating equations that included all symptoms, adjusted for age group, modelled separately by sex.

The results of the principal components analysis are shown in S2 Table. We found some clusters of symptoms, the first one containing fever (0.74), muscle ache (0.63) and a negative loading for runny nose (-0.43) indicating that this often did not occur in conjunction with fever and muscle ache. The second cluster contained cough (0.70), loss of smell or taste (0.47), and shortness of breath (0.62). Lastly, throat ache did not strongly cluster with any other symptom, but the factor analysis showed that people who report throat ache often did not report a runny nose.

## Discussion

Cough, fever, loss of smell or taste, and muscle ache at initial presentation were strongly associated with a positive test for SARS-CoV-2 in symptomatic people of the general population who were tested. A runny nose, shortness of breath, and throat ache were more associated with a negative test result in this study population. Although loss of smell or taste was a relatively specific symptom for positive SARS-CoV-2 tests, its prevalence was low amongst those with a positive test result. Runny nose and throat ache were the most prevalent symptoms, but they were more prevalent among those who tested negative than those who tested positive. Fever and muscle ache often seemed to cluster together, as did cough, loss of smell or taste, and shortness of breath.

### Strengths and limitations

The routing on the online test request application has changed several times during the study period. These changes in part followed national testing guidelines, such as that until December 1^st^, 2020, requesting a test was not allowed for a person currently having no symptoms. Starting December 1^st^, testing for asymptomatic individuals was allowed, when they were identified as a contact of a confirmed case by the contact tracing team or if they received a notification on the Covid tracker app. In these cases, making an appointment for testing was only possible by telephone call, and not through the online application. This might have prevented some people from making a testing appointment, potentially leading to underreporting. Conversely, others might have falsely reported non-existent symptoms, just in order to be allowed to request a test, leading to potential overreporting. Unfortunately, we do not know how systematically symptoms were assessed. Lastly, with more research on COVID-19/SARS-CoV-2 infections, our knowledge of the symptoms that are potentially important in identifying new cases of SARS-CoV-2 infection, especially with the spread of new variants, has advanced [28, 29]. Some of these symptoms, such as headache, fatigue, or diarrhoea were not included in the list of symptoms used in the Dutch data system. Therefore, our data might not show the complete picture of symptoms related to test outcome.

We only have data on people who requested a SARS-CoV-2 test, but not all symptomatic people requested a test. There might be several reasons why people might choose not to get tested, such as social or economic drawbacks of potentially having to self-isolate [30]; perceived reliability of tests, perceived discomfort of specimen collection [31]; views on testing policy, or trust in health experts’ advice [32]; or having only mild symptoms [33]. With such a variety of reasons, it is difficult to estimate whether significant selection bias caused by excluding asymptomatic people might have influenced our results, and to what extent these results are generalizable to the broader population. In addition, we do not have data on symptom severity, and therefore cannot make inferences about whether more severe symptoms are more predictive of a positive test result.

Strengths of this study include the large sample size, which means that the models have a high degree of statistical power which enables the detection of even very small effects. This does come with the caveat that some associations might be statistically significant, even though they might not be clinically relevant. Other strengths include the completeness of the records and the representativeness of the population as a result of using comprehensive data from the free-of-charge PHS testing facility, and the low risk of recall bias for symptoms as they were assessed at the moment of making a test appointment.

### Interpretation of results

The finding that loss of smell or taste was strongly associated with a positive SARS-CoV-2 test was not surprising, as this is a quite specific symptom that is not commonly associated with highly prevalent respiratory illnesses such as influenza or the common cold, although loss of smell might be associated with chronic rhinitis [34, 35]. However, the prevalence of loss of smell or taste among those who tested positive is still relatively low (19.2%), which means that the absence of this symptom is not useful to rule out SARS-CoV-2 infection.

The fact that a runny nose and throat ache were associated with a negative SARS-CoV-2 test result was also not surprising, given that these are symptoms commonly reported for influenza, common cold, or allergic rhinitis [11–13]. Therefore, even though in our sample of people with a positive test result, runny nose and throat ache were amongst the most commonly reported symptoms, they are poor predictors for SARS-CoV-2 infection. However, the incidence of influenza in the Netherlands in 2020 was very low or even non-existent compared to previous years, with about 20 consults per 100,000 GP patients per week, compared to a range of 6 up to 120 in the preceding years [36].

The finding that shortness of breath was not associated with a positive test was somewhat surprising, given that it is commonly reported in those with confirmed SARS-CoV-2 infection, with estimates of the prevalence ranging from 15% to 23% [7, 8]. However, dyspnoea may occur relatively late in the course of the infection [37]. In those who tested positive in our sample, the median time between symptom onset and applying for a test (at which time the symptoms were assessed) was only 57 hours [IQR = 33–88] [38], which would be too soon for dyspnoea to occur in the majority of patients.

Muscle ache was also found to be strongly associated with a positive test for SARS-CoV-2. Muscle ache is also commonly associated with influenza and other upper respiratory tract infections [12]. The main pathway from infection to muscle ache, shared between influenza and SARS-CoV-2, is through the release of cytokines. However, there might be alternative pathways specific to SARS-CoV-2, such as the virus entering via the angiotensin-converting enzyme 2 (ACE 2) receptors [39], or entering the central nervous system through the olfactory nerve [40].

Looking at the combined results, our findings seem to be mostly in line with those from other studies. Particularly the positive associations for cough, fever, loss of smell or taste with a positive SARS-CoV-2 status have been widely reported elsewhere [8, 20–22, 28, 41, 42]. Our finding of a strong association for muscle ache is supported by only one study [21], whereas in others the association between muscle ache and SARS-CoV-2 was either unclear, non-significant, or it was not assessed. For runny nose, most studies do not report a strong association with SARS-CoV-2 [20–22, 42], whereas for shortness of breath and sore throat there was mixed evidence. In contrast, we found very significant negative associations with SARS-CoV-2 infection for all three.

The performance of the overall prediction model is poor or fair at best, with an AUC of 0.72. This indicates that even though we found some symptoms to be significantly associated with a positive test result, the full model is not very good at distinguishing between positive and negative test results. This is also seen in the NPV and PPV values. The negative predictive values are fairly high (85.1% to 90.6%), but similar to the overall percentage of negative tests, meaning the absence of these symptoms is not really useful to rule out SARS-CoV-2 infection. Moreover, the positive predictive values were quite low (10.0% to 26.6%), indicating that the presence of these symptoms is not a good predictor of a positive rest result.

For some symptoms we saw some slight changes over time in the strength of their association with the test result. For loss of smell or taste it declined slightly, whereas for cough it increased slightly. These differences may partly be explained by a small number of positive tests in the early months, leading to lower statistical power and therefore less accurate estimates of effect sizes compared to the later months. The increasing association for cough might be related to the very low incidence of influenza in autumn [36], for which these are common symptoms. None of the other identified studies examined changes over time, therefore we cannot compare our results on this matter. However, one factor that may influence this is the arrival of new variants of SARS-CoV-2 in the Netherlands late in 2020. Early data showed that the Alpha variant might be associated less with loss of smell or taste, and more with cough, muscle ache, and throat ache [29]. But overall, our results show that the association between most individual symptoms and test result did not change much over time, despite the emergence of the Alpha and Delta variants.

### Implications

Symptoms play a large part in the public awareness and communication surrounding COVID-19, e.g. in terms of when to apply for a test, when to self-isolate, and when to avoid public places. Therefore, it is very important to know which symptoms are most predictive of an infection, and use the correct set of symptoms in public health communication. For instance in the UK, the NHS COVID-19 app now includes a symptom tool to guide when people should self-isolate and get tested, based on cough, fever, and loss of smell or taste [43]. Some of the symptoms that are currently emphasized (in the Netherlands), such as runny nose or throat ache are, although prevalent in those with a SARS-CoV-2 infection, not associated with infection. On the other hand, muscle ache was indicative of a positive test result. Therefore it would seem wise to emphasize muscle ache more in COVID-19 related communication in the Netherlands, in addition to cough, fever, and loss of smell or taste.

It is important to note that the association between symptoms and test result is likely to vary with specific populations. Therefore one overall SARS-CoV-2 prediction model might not be feasible, but population-specific models should be considered. Even within the multi-ethnic Amsterdam population, some differences in SARS-CoV-2 antibody prevalence were found between the largest ethnic groups, and strikingly 58.7% of people in the highest prevalence group were asymptomatic [44].

In a situation where testing capacity is limited, or significant barriers to testing exist, it would be helpful to have a screening tool to determine which people are at highest risk of being infected with SARS-CoV-2 to prioritize them for testing. Moreover, in a post-pandemic situation where nationwide disease prevention policies are scaled back, a simple screening tool could still be useful. In localized outbreaks, such a tool could help to determine when to use more specific diagnostic test and when to consider taking localized disease control measures to prevent the outbreak from spreading. The set of symptoms that we found to be associated with a positive test result in this paper combined with several symptoms reported elsewhere, and potentially more demographic information, could be a starting point for such a screening tool.

## Supporting information

S1 Table The number of symptoms reported by people requesting a SARS-CoV-2 test overall and by SARS-CoV-2 test result, from June 2020 through August 2021, Amsterdam region, the Netherlands. P-value for difference in number of symptoms between negative and positive test result from chi-square test is < 0.001.
(DOCX)

S2 Table Results of the principal components analysis of COVID-19 symptoms. Rotation Method: Varimax with Kaiser Normalization. Only factor loadings >0.30 or <-0.30 are shown.
(DOCX)

S1 Fig Flowchart of exclusions and records included in the analysis, Amsterdam region, the Netherlands, June 2020-August 2021. * This includes records with indeterminate or inconclusive test results.
(DOCX)

S2 Fig The prevalence of each symptom per month, from June 2020 through August 2021, the Amsterdam region, the Netherlands. (DOCX)
