# Supplementary material for: Self-reported symptoms as predictors of SARS-CoV-2 infection in the general population living in the Amsterdam region, the Netherlands
Source: PLoS One. 2022 Jan 28;17(1):e0262287. doi: 10.1371/journal.pone.0262287 (PMC8797231; doi:10.1371/journal.pone.0262287)
Supplement: S1 Table — P-value for difference in number of symptoms between negative and positive test result from chi-square test is < 0.001. (DOCX) [file pone.0262287.s001.docx]

**S1 Table:** The number of symptoms reported by people requesting a SARS-CoV-2 test overall and by SARS-CoV-2 test result, from June 2020 through August 2021, Amsterdam region, the Netherlands

|  | **Total** | | **Negative** | | **Positive** | |
| --- | --- | --- | --- | --- | --- | --- |
| **Number of symptoms** | **N** | **%** | **N** | **%** | **N** | **%** |
| 1 | 258,137 | 33.4 | 234,799 | 34.5 | 23,338 | 25.2 |
| 2 | 265,238 | 34.3 | 236,612 | 34.7 | 28,626 | 31.0 |
| 3 | 159,559 | 20.6 | 138,104 | 20.3 | 21,455 | 23.2 |
| 4 | 59,525 | 7.7 | 48,531 | 7.1 | 10,994 | 11.9 |
| 5 | 21,776 | 2.8 | 16,606 | 2.4 | 5,170 | 5.6 |
| 6 | 7,325 | 0.9 | 5,148 | 0.8 | 2,177 | 2.4 |
| 7 | 2,120 | 0.3 | 1,394 | 0.2 | 726 | 0.8 |

P-value for difference in number of symptoms between negative and positive test result from chi-square test is < 0.001.
