# Supplementary material for: Self-reported symptoms as predictors of SARS-CoV-2 infection in the general population living in the Amsterdam region, the Netherlands
Source: PLoS One. 2022 Jan 28;17(1):e0262287. doi: 10.1371/journal.pone.0262287 (PMC8797231; doi:10.1371/journal.pone.0262287)
Supplement: S2 Table — Rotation Method: Varimax with Kaiser Normalization. Only factor loadings >0.30 or <-0.30 are shown. (DOCX) [file pone.0262287.s002.docx]

**S2 Table:** Results of the principal components analysis of COVID-19 symptoms

|  | **Factor 1** | **Factor 2** | **Factor 3** |
| --- | --- | --- | --- |
| Cough |  | 0.70 |  |
| Fever | 0.74 |  |  |
| Loss of smell or taste |  | 0.47 |  |
| Muscle ache | 0.63 |  |  |
| Runny nose | -0.43 |  | -0.62 |
| Shortness of breath |  | 0.62 |  |
| Throat ache |  |  | 0.79 |

Rotation Method: Varimax with Kaiser Normalization.

Only factor loadings >0.30 or <-0.30 are shown.
