# Supplementary material for: Self-reported symptoms as predictors of SARS-CoV-2 infection in the general population living in the Amsterdam region, the Netherlands
Source: PLoS One. 2022 Jan 28;17(1):e0262287. doi: 10.1371/journal.pone.0262287 (PMC8797231; doi:10.1371/journal.pone.0262287)
Supplement: S1 Fig — * This includes records with indeterminate or inconclusive test results. (DOCX) [file pone.0262287.s003.docx]

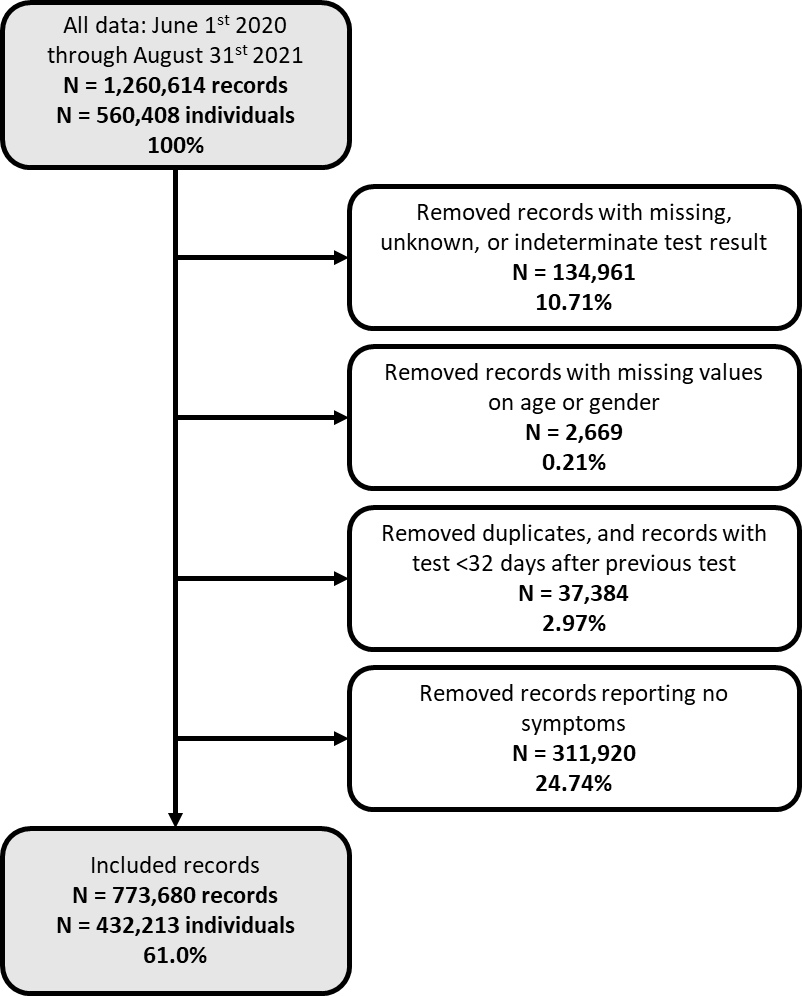


**S1 Fig:** Flowchart of exclusions and records included in the analysis, Amsterdam region, the Netherlands, June 2020 – August 2021.
