# Supplementary material for: Self-reported symptoms as predictors of SARS-CoV-2 infection in the general population living in the Amsterdam region, the Netherlands
Source: PLoS One. 2022 Jan 28;17(1):e0262287. doi: 10.1371/journal.pone.0262287 (PMC8797231; doi:10.1371/journal.pone.0262287)
Supplement: S2 Fig — (DOCX) [file pone.0262287.s004.docx]

**S2 Fig:** The prevalence of each symptom per month, from June 2020 through August 2021, the Amsterdam region, the Netherlands.

The area with white background indicates the period when the wild-type SARS-CoV-2 was dominant, the area shaded in yellow indicates the period during which the alpha-variant became dominant, and the area shaded in blue indicates the period during which the delta-variant became dominant.
